# Tratamento com Patisirana na Subpopulação Brasileira do Estudo de Fase 3 APOLLO-B em Amiloidose por Transtirretina com Cardiomiopatia: Análise Post Hoc

**DOI:** 10.36660/abc.2024568

**Published:** 2025-04-08

**Authors:** Claudio Tinoco Mesquita, Pedro Schwartzmann, Edileide Barros Correia, Marcus V. Simões, Andreia Biolo, Daniel Rodriguez Duque, Patrick Y. Jay, Fábio Fernandes

**Affiliations:** 1 Hospital Pró-Cardíaco Rio de Janeiro RJ Brasil Hospital Pró-Cardíaco, Rio de Janeiro, RJ – Brasil; 2 Universidade Federal Fluminense Niterói RJ Brasil Universidade Federal Fluminense, Niterói, RJ – Brasil; 3 Unimed Ribeirão Preto Ribeirão Preto SP Brasil Unimed Ribeirão Preto, Ribeirão Preto, SP – Brasil; 4 Instituto Dante Pazzanese de Cardiologia São Paulo SP Brasil Instituto Dante Pazzanese de Cardiologia, São Paulo, SP – Brasil; 5 Universidade de São Paulo Hospital das Clínicas Faculdade de Medicina de Ribeirão Preto Ribeirão Preto SP Brasil Universidade de São Paulo Hospital das Clínicas da Faculdade de Medicina de Ribeirão Preto, Ribeirão Preto, SP – Brasil; 6 Universidade Federal do Rio Grande do Sul Porto Alegre RS Brasil Universidade Federal do Rio Grande do Sul, Porto Alegre, RS – Brasil; 7 Alnylam Pharmaceuticals Inc Cambridge Massachusetts EUA Alnylam Pharmaceuticals Inc, Cambridge, Massachusetts – EUA; 8 Hospital das Clínicas Faculdade de Medicina Universidade de São Paulo São Paulo SP Brasil Instituto do Coração do Hospital das Clínicas da Faculdade de Medicina da Universidade de São Paulo, São Paulo, SP – Brasil

**Keywords:** Amiloidose, Transtirretina, Cardiomiopatias, RNA Interferente Pequeno, Ensaio Clínico

## Abstract

**Fundamento:**

A patisirana reduziu rapidamente a transtirretina e preservou a capacidade funcional em pacientes com amiloidose por transtirretina com cardiomiopatia (ATTR-CM) no estudo Fase 3 APOLLO-B (NCT03997383).

**Objetivos:**

Avaliar a eficácia e segurança da patisirana (análise post hoc) na subpopulação brasileira do APOLLO-B.

**Métodos:**

Pacientes foram randomizados 1:1 para patisirana 0,3 mg/kg ou placebo uma vez a cada 3 semanas por 12 meses. O desfecho primário foi a alteração em relação ao período basal (ARPB) na capacidade funcional (teste de caminhada de 6 minutos [6MWT]) no mês 12. Desfechos secundários incluíram ARPB no mês 12 do escore *Kansas City Cardiomyopathy Questionnaire-Overall Summary (KCCQ-OS)*. Desfechos exploratórios incluíram ARPB em biomarcadores cardíacos e na escala de Perugini durante cintilografia com 99m-Tecnécio pirofosfato.

**Resultados:**

Quarenta e dois pacientes foram incluídos no Brasil (patisirana, n=20; placebo, n=22). Patisirana demonstrou benefício no 6MWT e nos escores KCCQ-OS vs. placebo; ARPB (intervalo de confiança [IC] de 95%) no 6MWT (mediana) e escores KCCQ-OS (média dos mínimos quadrados) foram -2,0 m (-58,5; 42,9) e 9,37 (1,93; 16,81) pontos com patisirana vs. -30,1 m (-72,2; 3,5) e 2,62 (-4,68; 9,92) pontos para o placebo. Para biomarcadores cardíacos, a alteração média da razão em relação ao período basal (IC 95%) para peptídeo natriurético tipo B pró-hormonal N-terminal e troponina I foi de 1,31 (1,06; 1,61) e 1,12 (0,94; 1,34) para patisirana e 1,71 (1,39; 2,10) e 1,28 (1,08; 1,53) para placebo, respectivamente. A escala de Perugini melhorou em 11/18 (61,1%) pacientes e 0/10 pacientes com patisirana e placebo, respectivamente. Não houve mortes no grupo patisirana vs. 3 mortes no grupo placebo.

**Conclusão:**

A eficácia e a segurança da patisirana em pacientes brasileiros com ATTR-CM foram consistentes com as da população global do APOLLO-B. Os achados são descritivos devido ao pequeno número de pacientes.

## Graphical abstract

**Figura Central:**
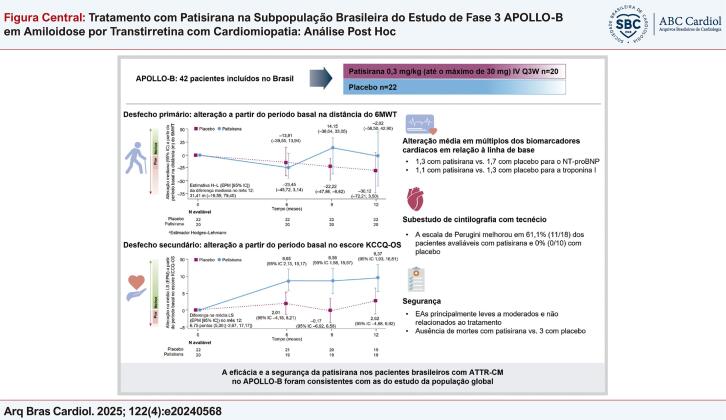
: Tratamento com Patisirana na Subpopulação Brasileira do Estudo de Fase 3 APOLLO-B em Amiloidose por Transtirretina com Cardiomiopatia: Análise Post Hoc 6MWT: teste de caminhada de 6 minutos; ATTR-CM: amiloidose por transtirretina com cardiomiopatia; EAs: eventos adversos; EPM: erro padrão da media; H–L: Hodges–Lehmann; IC: intervalo de confiança; IV: intravenosa; KCCQ-OS: Kansas City Cardiomyopathy Questionnaire-Overall Summary; LS: mínimos quadrados; NT-proBNP: porção N-terminal do pró-peptídeo natriurético tipo B; Q3W: uma vez a cada 3 semanas.

## Introdução

A amiloidose mediada por transtirretina (ATTR) é uma doença rara, rapidamente progressiva, debilitante e potencialmente fatal.^1^ A transtirretina (TTR) mal enovelada forma fibrilas amiloides tóxicas que se depositam em vários órgãos e sistemas, incluindo coração, nervos, trato gastrointestinal e sistema musculoesquelético.^1-3^ A ATTR pode ser hereditária (hATTR, também conhecida como ATTRv), onde os pacientes herdam variantes no *TTR* que desestabilizam a proteína TTR, causando sua dissociação e má conformação, ou do tipo selvagem (wtATTR), onde a TTR do tipo selvagem mal enovelada se acumula como depósitos amiloides em adultos mais velhos.^1^ Clinicamente, a hATTR se apresenta como polineuropatia, cardiomiopatia ou comumente ambas.^1,4,5^ Por outro lado, a wtATTR manifesta-se principalmente como cardiomiopatia, mas a polineuropatia também pode estar presente.^2,5^

Dados do registro internacional *Transthyretin Amyloidosis Outcomes Survey* (THAOS) mostram que a maioria dos pacientes sintomáticos com ATTR no Brasil apresenta manifestações neurológicas e do sistema gastrointestinal, e aproximadamente 30% têm envolvimento cardíaco.^6^ Cerca de 25% dos pacientes brasileiros com ATTR sintomática no registro THAOS foram diagnosticados erroneamente, e mais de um terço sofreu atraso no diagnóstico superior a um ano, o que pode ter resultado em atraso no início do tratamento.^6^ Sem intervenção, o acúmulo contínuo de amiloide no coração promove a progressão da cardiomiopatia e agrava as manifestações cardíacas associadas.^7^ Consequentemente, a ATTR com cardiomiopatia (ATTR-CM) apresenta um curso grave e progressivo, colocando os indivíduos afetados em alto risco de hospitalização cardiovascular e morte.^7^ A sobrevida mediana após o diagnóstico em pacientes não tratados é limitada: 2,5 anos para a hATTR com a mutação *TTR* Val122Ile (ou pV142I) e 3,6 anos para a wtATTR.^8^ As diretrizes de consenso da Sociedade Brasileira de Cardiologia destacam a importância do diagnóstico e tratamento precoces da ATTR-CM.^9^ No entanto, as opções terapêuticas disponíveis para essa condição ainda são limitadas.

A patisirana, uma terapia de RNA de interferência (RNAi) formulada em uma nanopartícula lipídica, tem como alvo o mRNA hepático da TTR e reduz rapidamente os níveis circulantes das proteínas TTR do tipo selvagem e variante.^10^ Essa terapia foi aprovada para o tratamento de hATTR com polineuropatia.^11,12^ A patisirana não é aprovada para o tratamento de ATTR-CM nos EUA, mas foi aprovada recentemente no Brasil pela *Agência Nacional de Vigilância Sanitária* (ANVISA), e a *Agence nationale de sécurité du médicament et des produits de santé* (ANSM) concedeu aprovação para o uso compassivo de patisirana na França para pacientes com ATTR-CM com falha ao tratamento com tafamidis 61 mg. No estudo de Fase 3 APOLLO-B (NCT03997383), a patisirana preservou a capacidade funcional, o estado de saúde e a qualidade de vida durante 12 meses em comparação ao placebo em pacientes com ATTR-CM (hATTR e wtATTR).^13^ Análises exploratórias também mostraram melhora nos biomarcadores cardíacos e na estrutura e função ventricular esquerda (VE) em comparação com o placebo.^13^ Esta análise post hoc avaliou a eficácia e segurança da patisirana na subpopulação brasileira do APOLLO-B. Também avaliamos a cintilografia com o marcador ósseo de 99m-Tecnécio, uma técnica diagnóstica não invasiva para ATTR-CM, para entender melhor o impacto da terapia na captação cardíaca deste marcador.

## Métodos

### Desenho do estudo

O APOLLO-B é um estudo internacional, de Fase 3, randomizado e multicêntrico, que avalia a patisirana em pacientes com ATTR-CM (hATTR e wtATTR), compreendendo um período de 12 meses, duplo-cego e controlado por placebo, e um período aberto de extensão em andamento (OLE; NCT02510261). O estudo está sendo conduzido em 21 países da América do Norte, América Latina, Europa e Ásia-Pacífico. Detalhes completos do desenho do estudo foram publicados.^13^

Resumidamente, os pacientes foram randomizados na proporção de 1:1 para receber patisirana (0,3 mg/kg até um máximo de 30 mg) ou placebo por via intravenosa (IV) uma vez a cada 3 semanas (Q3W) por 12 meses. Todos os pacientes em ambos os braços de tratamento receberam pré-medicação padrão 60 minutos antes da infusão para reduzir o potencial de uma reação relacionada à infusão com a patisirana. Os pacientes que completaram o período duplo-cego de 12 meses foram elegíveis para receber patisirana 0,3 mg/kg IV Q3W no período OLE de 36 meses em andamento.

O protocolo do estudo e seus aditivos foram revisados e aprovados pelo Comitê de Revisão Institucional ou Comitê de Ética Independente em cada centro. O estudo foi conduzido de acordo com todos os requisitos regulatórios aplicáveis, as diretrizes atuais de Boas Práticas Clínicas e os princípios originários da Declaração de Helsinque. Todos os pacientes forneceram consentimento informado por escrito antes da participação.

### Pacientes

Os critérios completos de inclusão e exclusão foram publicados.^13^ Os pacientes tinham entre 18 e 85 anos; com diagnóstico de ATTR-CM (hATTR ou wtATTR), definido como deposição de amiloide TTR em biópsia de tecido ou preenchendo critérios diagnósticos validados sem biópsia (ou seja, captação cardíaca de Grau 2 ou 3 na cintilografia com tecnécio em pacientes que não têm gamopatia monoclonal);^14^ evidência de envolvimento cardíaco por ecocardiografia, com espessura da parede septal interventricular em diástole final >12 mm; e histórico médico de insuficiência cardíaca. Os pacientes não receberam tratamento prévio com tafamidis ou foram permitidos ter recebido tafamidis por ≥6 meses no período basal conforme a bula local e dose aprovada, com progressão da doença conforme determinada pelo investigador.

Os pacientes foram excluídos se classificados como Classe III da *New York Heart Association* (NYHA) e estágio 3 de ATTR (definido como porção N-terminal do pró-peptídeo BNP [NT-proBNP] >3000 ng/L, concomitantemente com uma taxa de filtração glomerular estimada <45 mL/min/1,73 m^2^)^15^ Classe IV da NYHA; distância no teste de caminhada de 6 minutos (6MWT) <150 m; escore de incapacidade por polineuropatia (PND) >2; e outras cardiomiopatias não relacionadas à TTR.

### Desfechos

O desfecho primário foi a alteração em relação ao período basal na capacidade funcional medida pelo 6MWT no Mês 12 para patisirana vs. placebo. Os desfechos secundários pré-especificados avaliados na subpopulação brasileira incluíram: alteração do período basal até o Mês 12 no escore geral do *Kansas City Cardiomyopathy Questionnaire-Overall Summary (KCCQ-OS*) e nos escores dos domínios;^16^ desfecho composto de mortalidade por todas as causas, frequência de hospitalizações por todas as causas e consultas urgentes por insuficiência cardíaca ao longo de 12 meses. Os desfechos exploratórios incluíram a mudança nos biomarcadores cardíacos (NT-proBNP e troponina I) nos Meses 3, 6, 9 e 12, e a escala de Perugini avaliada pela captação cardíaca durante a cintilografia com 99m-Tecnécio com marcadores ósseos. A cintilografia com 99m-Tecnécio pirofosfato foi realizada em um subgrupo planejado de pacientes. Os efeitos farmacodinâmicos foram avaliados pelas mudanças nos níveis séricos de TTR do valor basal até o Mês 12. Os desfechos de segurança, incluindo eventos adversos (classificados de acordo com o Sistema de Classe de Órgãos e Termo Preferido da versão 23.0 do Dicionário Médico para Assuntos Regulatórios), parâmetros laboratoriais clínicos e sinais vitais foram monitorados ao longo do estudo.

### Análises estatísticas

Para o desfecho primário, o tamanho mediano do efeito do tratamento com intervalo de confiança de 95% foi estimado utilizando o estimador de Hodges–Lehmann; dados ausentes foram imputados conforme descrito anteriormente.^13^ A mediana foi usada devido à não normalidade do desfecho de 6MWT. As alterações a partir do valor basal no Mês 12 no KCCQ-OS e nos escores dos componentes foram avaliadas usando um modelo de efeitos mistos para medidas repetidas (MEMMR) ajustado para o KCCQ-OS basal como covariável contínua e termos de efeito fixo (braço de tratamento, visita, tafamidis basal, subgrupo [Brasil vs. fora do Brasil], interação tratamento-por-visita, interação tratamento-por-subgrupo, interação visita-por-subgrupo e interação tratamento-por-visita-por-subgrupo). A mudança do valor basal nos biomarcadores cardíacos (NT-proBNP e troponina I) foi avaliada com um modelo MEMMR, com valores basais transformados para logaritmo como covariável contínua e os mesmos termos de efeito fixo da análise do KCCQ-OS. Permitindo o tamanho da amostra, as análises seguiram os modelos pré-especificados para análise de subgrupos. Os modelos MEMMR foram ajustados usando todos os participantes do estudo, não apenas os participantes brasileiros, e foram as interações de subgrupos dentro dos modelos que permitiram a estimativa de efeitos específicos para a subpopulação brasileira.

Um subgrupo de pacientes no APOLLO-B participou de um estudo planejado de imagem por tomografia computadorizada por emissão de fóton único com tecnécio usando marcadores ávidos por osso. O grau de Perugini foi avaliado no período basal e no Mês 12 (grau 0 = ausência de captação cardíaca; 1 = captação cardíaca leve menor que osso; 2 = captação cardíaca moderada igual ao osso ou com captação óssea levemente atenuada; 3 = captação cardíaca alta maior que osso ou com redução acentuada na captação óssea).^14^ A mudança do valor basal até o Mês 12 na escala de Perugini foi resumida descritivamente.

As características demográficas e basais, mortes, frequência de hospitalizações por qualquer causa, consultas urgentes por insuficiência cardíaca na população geral e dados de segurança foram resumidos descritivamente. Eventos devido à COVID-19 foram excluídos da análise. As análises de eficácia e segurança foram conduzidas na subpopulação brasileira do conjunto completo de análise (ou seja, todos os pacientes randomizados que receberam qualquer quantidade do medicamento do estudo) para o período duplo-cego do estudo.

As análises farmacodinâmicas foram conduzidas em todos os pacientes que receberam ≥1 dose completa do medicamento do estudo e tinham uma linha de base avaliável e ≥1 medição sérica de TTR pós-linha de base avaliável. Os percentuais das alterações a partir dos períodos basais nos níveis séricos de TTR foram resumidos descritivamente.

Todas as análises descritivas buscaram resumir variáveis contínuas e normais por meio de médias e desvios padrão, variáveis contínuas e não normais por meio de medianas e intervalos interquartis, e variáveis categóricas por meio de frequências e porcentagens. A normalidade foi avaliada visualmente através de histogramas; as distribuições de dados que apresentavam assimetria ou que continham *outliers* foram consideradas não-normais. O SAS versão 9.4 foi usado para realizar as análises estatísticas.

## Resultados

### Pacientes

Um total de 360 pacientes foram incluídos no APOLLO-B entre outubro de 2019 e maio de 2021. Destes, 42 pacientes foram incluídos em 6 centros no Brasil; dos quais 39 completaram a visita do Mês 12 (Figura S1 dos Materiais Suplementares). As características demográficas e clínicas basais para a subpopulação brasileira foram geralmente comparáveis entre os grupos de tratamento, exceto por menores proporções numéricas de homens e pacientes com wtATTR no grupo patisirana em comparação com o placebo (Tabela 1). A mediana (intervalo) de idade foi de 73 (51–85) anos na subpopulação brasileira. Nenhum paciente estava recebendo tafamidis no período basal, mas todos os pacientes estavam recebendo medicamentos para doenças cardíacas concomitantes (veja a Tabela S1 dos Materiais Suplementares para detalhes). Vinte e três (54,8%) pacientes tinham wtATTR e 19 (45,2%) tinham hATTR. Entre aqueles com hATTR, havia 5 variantes de TTR; a mais comum sendo V122I (Tabela 1). Um fenótipo misto com cardiomiopatia e polineuropatia foi relatado em 17 (89,5%) pacientes com hATTR (veja a Tabela S1 dos Materiais Suplementares para demografia e características clínicas basais).

**Tabela 1:** – Características demográficas e clínicas no período basal

	Patisirana (n=20)	Placebo (n=22)
Idade (anos), mediana (IIQ)*	73 (69–82)	73 (71–78)
**Gênero masculino, n (%)**	14 (70,0)	20 (90,9)
**Raça, n (%)^†^**		
Branco	12 (60,0)	16 (72,7)
Negro/Afro-americano	6 (30,0)	5 (22,7)
Asiático	0	1 (4,5)
Outro	1 (5,0)	0
Não relatado	1 (5,0)	0
**wtATTR, n (%)**	7 (35,0)	16 (72,7)
Diagnosticado por biópsia^‡^	0	4 (25,0)
Diagnosticado por cintilografia com tecnécio^‡^	7 (100,0)	15 (93,8)
**hATTR, n (%)**	13 (65,0)	6 (27,3)
Diagnosticado por biópsia^‡^	2 (15,4)	2 (33,3)
Diagnosticado por cintilografia com tecnécio^‡^	12 (92,3)	6 (100,0)
**hATTR com fenótipo misto de cardiomiopatia e polineuropatia, n (%)^§^**	13 (100,0)	4 (66,7)
**Variantes TTR, n (%)^§^**		
I68L	0	1 (16,7)
T60A	3 (23,1)	1 (16,7)
V122I	9 (69,2)	3 (50,0)
V122L	0	1 (16,7)
V30M	1 (7,7)	0
**Tempo desde o diagnóstico de ATTR (anos), mediana (intervalo)**	0,6 (0,1–1,8)	0,3 (0,1–1,6)
**Uso de tafamidis no período basal, n (%)**	0	0
**Estágio de ATTR, n (%)^¶^**		
1	13 (65,0)	12 (54,5)
2	7 (35,0)	8 (36,4)
3	0	2 (9,1)
**Escore PND, n (%)**		
0: sem prejuízo	6 (30,0)	11 (50,0)
I: marcha preservada, com distúrbios sensitivos	13 (65,0)	11 (50,0)
II: comprometida, caminhando sem a necessidade de uma bengala ou muletas	1 (5,0)	0
**Classe NYHA, n (%)**		
I	0	1 (4,5)
II	20 (100,0)	20 (90,9)
III	0	1 (4,5)
**Distância no 6MWT (m), mediana (IIQ)**	360,4 (323,8–379,1)	350,3 (300,0–434,7)
**KCCQ-OS (pontos), média (SD)**	59,7 (22,4)	63,2 (23,2)
**Nível de NT-proBNP (ng/L), mediana (IIQ)**	1832,5 (904,5–3457,0)	2155,0 (1030,0–2911,0)
**Nível de Troponina I (ng/L), mediana (IIQ)**	64,2 (38,6–122,5)	78,0 (27,6–125,7)
**eGFR (mL/min/1,73m^2^), mediana (IIQ)**	61,5 (54,5–72,5)	55,5 (39,0–72,0)
**Creatinina (μmol/L), mediana (IIQ)**	101,5 (84,0–124,0)	115,0 (80,0–150,0)
**Histórico médico, n (%)**		
Diabetes mellitus	7 (35,0)	6 (27,3)
Hipertensão	12 (60,0)	11 (50,0)
**Medicações concomitantes, n (%)**		
Diuréticos	18 (90,0)	21 (95,5)
Antagonista dos receptores de mineralocorticoides	13 (65,0)	11 (50,0)
Betabloqueadores	5 (25,0)	11 (50,0)
IECA, BRA, ou IRAN	11 (55,0)	9 (40,9)
Inibidores SGLT2	3 (15,0)	4 (18,2)

*Idade na triagem; ^†^Autorrelatado; ^‡^O diagnóstico de ATTR pode ser confirmado por múltiplos métodos, portanto, a soma das porcentagens pode ser maior que 100; ^§^Porcentagem baseada no número de pacientes com hATTR; ^¶^Estágio 1 (menor risco) foi definido por nível de NT-proBNP ≤ 3000 pg/mL e eTFG de ≥45 mL/min/1,73 m^2^. Estágio 2 (risco intermediário) incluiu todos os pacientes que não atendiam aos critérios para os estágios 1 ou 3. Estágio 3 (maior risco) foi definido por NT-proBNP ≥3000 pg/mL e uma eTFG de <45 mL/min/1,73 m^2^. 6MWT: teste de caminhada de 6 minutos; IECA: inibidor da enzima conversora de angiotensina; BRA: bloqueador do receptor de angiotensina; IRAN: inibidor do receptor de angiotensina-neprilisina; ATTR: amiloidose por transtirretina; eTFG: taxa de filtração glomerular estimada; hATTR: amiloidose hereditária por transtirretina; IIQ: intervalo interquartil; KCCQ-OS: Questionário de Cardiomiopatia de Kansas City - Resumo Geral; NT-proBNP: peptídeo natriurético tipo B pró-hormonal N-terminal; NYHA: New York Heart Association; PND: incapacidade por polineuropatia; SD: desvio padrão; SGLT2: co-transportador de sódio-glicose 2; TTR: transtirretina; wtATTR: amiloidose por transtirretina do tipo selvagem.

## Eficácia

### Desfecho primário

**Figura 1:**
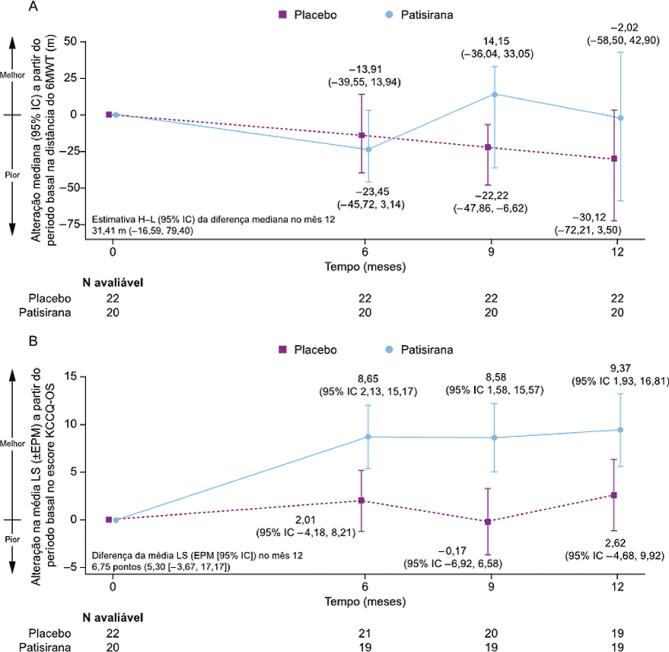
– Alteração a partir do período basal ao longo do tempo para (a) 6MWT e (B) KCCQ-OS. 6MWT: Teste de caminhada de 6 minutos; H–L: Hodges–Lehmann; IC: intervalo de confiança; KCCQ-OS: Kansas City Cardiomyopathy Questionnaire-Overall Summary; LS: mínimos quadrados; EPM: erro padrão da média.

Pacientes tratados com patisirana na subpopulação brasileira mostraram uma menor magnitude de declínio em relação ao valor basal na distância do 6MWT no Mês 12 em comparação com o placebo (Figura 1A).

### Desfechos secundários

Os escores KCCQ-OS aumentaram a partir do valor basal até o Mês 12 com patisirana vs. placebo (Figura 1B). As análises dos componentes favoreceram patisirana vs. placebo para todos os domínios (limitação física, sintomas totais, qualidade de vida e limitação social) (Figura S2 dos Materiais Suplementares). Uma proporção similar de pacientes nos grupos patisirana e placebo tiveram ≥1 evento composto (mortalidade por todas as causas ou hospitalização por todas as causas ou consulta urgente por insuficiência cardíaca; Tabela 2). Não houve mortes no grupo patisirana (Tabela 2).

**Tabela 2:** – Mortes por todas as causas, hospitalizações por todas as causas, ou consultas urgentes por insuficiência cardíaca*

	Patisirana (n=20) AP=20,9	Placebo (n=22) AP=21,6
**Paciente com ≥1 evento^†‡^, n (%) [n eventos]**	6 (30,0) [10]	6 (27,3) [11]
**Mortes por todas as causas, n (%) [n eventos]**	0	3 (13,6) [3]
Mortes relacionadas ao aparelho cardiovascular	0	2 (9,1) [2]
Mortes não relacionadas ao aparelho cardiovascular	0	1 (4,5) [1]
**Hospitalizações por todas as causas, n (%) [n eventos]**	5 (25,0) [9]	6 (27,3) [8]
Hospitalizações relacionadas ao aparelho cardiovascular	3 (15,0) [4]	4 (18,2) [5]
Hospitalizações não relacionadas ao aparelho cardiovascular	2 (10,0) [5]	3 (13,6) [3]
**Consultas urgentes por insuficiência cardíaca, n (%) [n eventos]**	1 (5,0) [1]	0

*Tempo de acompanhamento calculado usando o último dia no período duplo-cego de 12 meses para análise de eficácia; ^†^Desfecho composto de morte por todas as causas, hospitalizações por todas as causas ou visitas urgentes por insuficiência cardíaca; ^‡^Eventos devido à COVID-19 foram excluídos. AP: anos-paciente.

### Desfechos exploratórios

**Figura 2:**
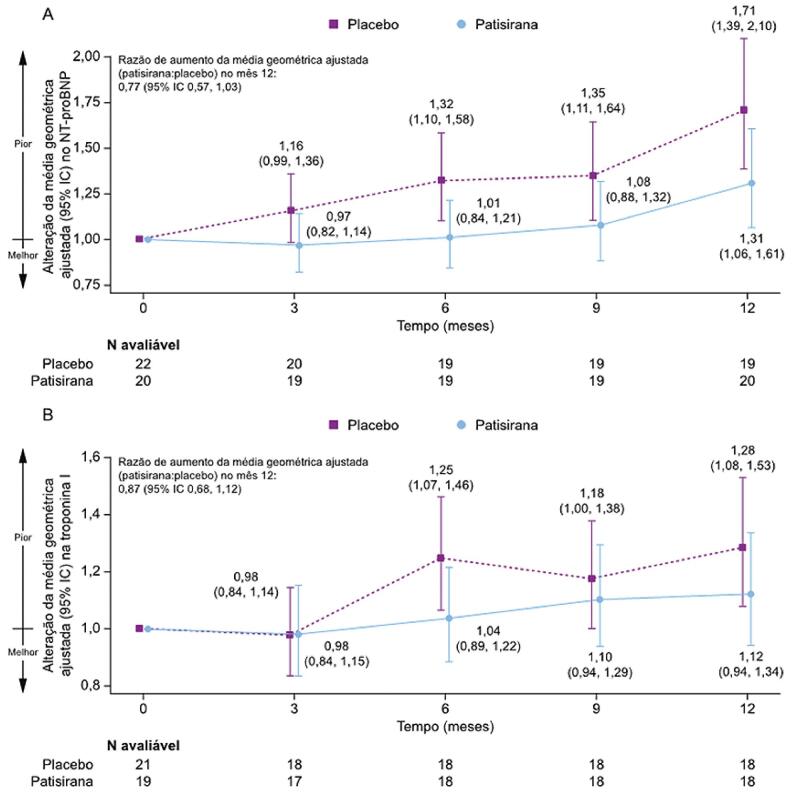
– Alteração da média geométrica ajustada a partir do período basal em (A) NT-proBNP e (B) troponina I. IC: intervalo de confiança: NT-proBNP: porção N-terminal do pró-peptídeo natriurético tipo B.

A mediana (intervalo interquartil [IIQ]) da mudança em relação ao valor basal no NT-proBNP no Mês 12 foi de 320,0 (3,5–1756,0) ng/L com patisirana e 2024,0 (–211,0–3231,7) ng/L com placebo. A razão (patisirana:placebo) da média geométrica ajustada da mudança em relação ao valor basal foi maior no grupo do placebo do que no grupo da patisirana (Figura 2A). Para troponina I, uma mediana (IIQ) da mudança em relação ao valor basal de 8,5 (-9,10–23,4) ng/L foi relatada no Mês 12 para o grupo patisirana em comparação com 16,5 (0–61,5) ng/L para placebo. A razão (patisirana:placebo) da média geométrica ajustada da mudança em relação ao valor basal foi maior no grupo do placebo do que no grupo da patisirana (Figura 2B).

**Figura 3:**
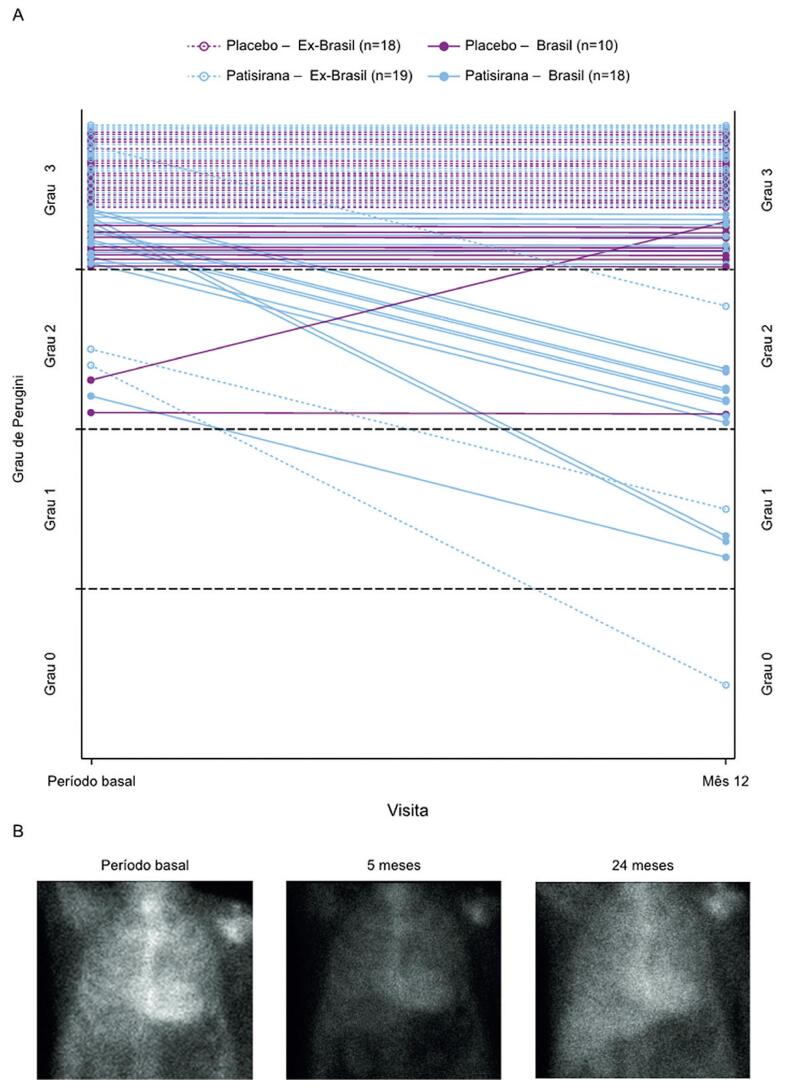
- Dados individuais de pacientes do Brasil e fora do Brasil para (A) escala de Perugini no período basal e no Mês 12, e (B) imagens representativas de cintilografia cardíaca com tecnécio-99m Pirofosfato para um paciente individual mostrando uma redução na captação miocárdica do marcador aos 5 e 24 meses após o início do tratamento com patisirana (conjunto de análise de tecnécio).

Trinta e cinco pacientes (patisirana, n=18; placebo, n=17) na subpopulação brasileira foram incluídos no subgrupo de avaliação por imagem. Vinte e oito pacientes (patisirana, n=18; placebo, n=10) foram avaliados com a escala de Perugini na linha de base e no Mês 12; 7 pacientes no grupo placebo não foram avaliados no Mês 12. Uma melhora na escala de Perugini no Mês 12 foi observada em 11 (61,1%) pacientes no grupo patisirana. Destes 11 pacientes, 3 melhoraram para o grau 1 de Perugini. Nenhum dos pacientes tratados com placebo mostrou melhora na escala de Perugini. Nenhum paciente no grupo patisirana mostrou aumento na escala de Perugini, enquanto 1 paciente (10,0%) no grupo placebo mostrou aumento na escala no Mês 12. Dados relacionados aos pacientes para a mudança em relação ao valor basal na escala de Perugini para pacientes na subpopulação brasileira e aqueles fora do Brasil são mostrados na Figura 3A, e imagens representativas são mostradas na Figura 3B.

### Farmacodinâmica

**Figura 4:**
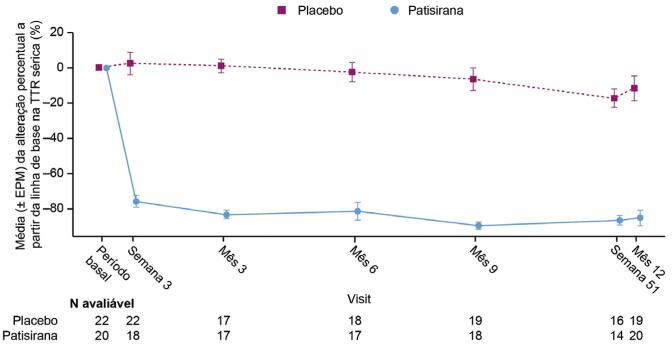
– Alteração percentual a partir do período basal na TTR sérica. Os níveis séricos da TTR foram analisados conforme previamente descrito.13 EPM: erro padrão da média; TTR: transtirretina.

Foi observada uma rápida e sustentada redução dos níveis séricos de TTR em pacientes tratados com patisirana (Figura 4). No mês 12, o tratamento com patisirana levou a uma redução média (erro padrão da média) percentual nos níveis séricos de TTR de 85,3% (4,5).

## Segurança

Uma proporção semelhante de pacientes do Brasil nos grupos patisirana e placebo teve eventos adversos durante o período duplo-cego de 12 meses (Tabela 3), com a maioria sendo de gravidade leve ou moderada e considerados não relacionados ao tratamento do estudo pelos investigadores. O evento adverso mais frequente foi insuficiência cardíaca (Tabela 3). Nenhum paciente do Brasil teve reações relacionadas à infusão. Eventos adversos graves estão apresentados na Tabela S3 dos Materiais Suplementares. Nenhum evento adverso resultou em descontinuações e ocorreram 3 mortes, todas no grupo placebo.

**Tabela 3:** – Visão geral dos eventos adversos durante o período de tratamento duplo-cego randomizado de 12 meses

Evento, n paciente (%)	Patisirana (n=20) AP=20,9^*^	Placebo (n=22) AP=21,5^*^
**≥1 EA (todas as causas)**	19 (95,0)	21 (95,5)
**EAs relacionados ao tratamento**	2 (10,0)	2 (9,1)
**EAs mais comuns (todas as causas)^†^**		
Insuficiência cardíaca	5 (25,0)	7 (31,8)
Insuficiência renal	3 (15,0)	2 (9,1)
Espasmos musculares	3 (15,0)	1 (4,5)
COVID-19	3 (15,0)	2 (9,1)
Constipação	2 (10,0)	4 (18,2)
Náusea	2 (10,0)	0
Sepse urinária	2 (10,0)	0
Desequilíbrio do sistema nervoso autônomo	2 (10,0)	0
Insônia	2 (10,0)	3 (13,6)
Hematúria	2 (10,0)	1 (4,5)
Fibrilação atrial	1 (5,0)	3 (13,6)
**EAGs (todas as causas)**	8 (40,0)	7 (31,8)
**EAGs relacionados ao tratamento**	0	0
**EAs cardíacos (CSO)**	7 (35,0)	10 (45,5)
**EAGs**	3 (15,0)	4 (18,2)
Fibrilação atrial	0	1 (4,5)
Insuficiência cardíaca	2 (10,0)	4 (18,2)
Taquiarritmia	1 (5,0)	0
**EAs Severos**	4 (20,0)	5 (22,7)
**EAs levando à descontinuação do tratamento**	0	0
**EAs relacionados ao tratamento levando à descontinuação do tratamento**	0	0
**Mortes**	0	3 (13,6)

Eventos adversos codificados usando a versão 23.0 do MedDRA. *Determinado usando o último dia de administração no período de 12 meses; ^†^ ≥10% para Termos Preferidos em qualquer braço de tratamento. EA: evento adverso; MedDRA: Dicionário Médico para Assuntos Reguladores; AP: anos-paciente; EAG: evento adverso grave; CSO: classe de sistema de órgãos.

## Discussão

Esta análise post hoc avaliou a eficácia e segurança da patisirana em pacientes com ATTR-CM (hATTR e wtATTR) da subpopulação brasileira do estudo APOLLO-B. Embora os resultados sejam descritivos, pois o estudo não foi desenhado para detectar efeitos específicos de tratamento neste subgrupo, eles são consistentes com os achados gerais do APOLLO-B. A rápida e sustentada redução da TTR circulante pela patisirana resultou em potencial melhoria na capacidade funcional, estado de saúde e qualidade de vida durante o período duplo-cego de 12 meses em comparação com o placebo, e foi bem tolerado em pacientes brasileiros com ATTR-CM. Um benefício potencial nos biomarcadores cardíacos e na captação miocárdica na cintilografia com traçadores ávidos por osso (grau de Perugini) também foi observado.

Uma diferença clinicamente significativa foi evidente com patisirana em termos de preservação da capacidade funcional, com a magnitude observada de declínio na distância do 6MWT no grupo patisirana sendo semelhante àquela esperada com o envelhecimento saudável.^17,18^ Uma tendência de melhoria no estado de saúde e qualidade de vida, baseada em uma diferença moderada a grande entre os grupos patisirana e placebo na mudança em relação ao valor basal nos escores KCCQ-OS no Mês 12, também foi evidente. Patisirana teve um efeito notável nos sintomas, com uma diferença em relação ao placebo de aproximadamente 12 pontos no domínio de sintomas do escore KCCQ, e um impacto correspondente nos domínios de qualidade de vida. Esses efeitos são relevantes para pacientes com ATTR na prática clínica, que frequentemente relatam sintomas debilitantes que afetam negativamente suas vidas diárias e bem-estar emocional.^19^

Algumas diferenças foram evidentes nos resultados na subpopulação brasileira em comparação com a população geral do estudo. Entre os pacientes tratados com patisirana, os escores KCCQ-OS não aumentaram na população geral do APOLLO-B na mesma extensão observada na subpopulação brasileira. Pacientes tratados com placebo no Brasil parecem mostrar um declínio menos rápido na capacidade funcional do que os pacientes na população geral do estudo. Além disso, o estado de saúde e a qualidade de vida foram mantidos no grupo placebo da subpopulação brasileira, enquanto houve uma diminuição (piora) nos escores entre os pacientes tratados com placebo na população geral do estudo. A razão para essas diferenças é desconhecida, mas pode estar relacionada ao manejo geral da insuficiência cardíaca ou a um efeito placebo.

Além de seus efeitos na capacidade funcional, estado de saúde e qualidade de vida, o tratamento com patisirana resultou em menor aumento nos biomarcadores cardíacos NT-proBNP e troponina I, em comparação com o placebo. NT-proBNP e troponina I são usados para avaliar manifestações cardíacas em pacientes com ATTR-CM, e níveis elevados estão associados a um envolvimento cardíaco mais grave e piores desfechos cardíacos e de mortalidade.^20,21^ A melhora nos níveis desses biomarcadores cardíacos observada em pacientes brasileiros com ATTR-CM no APOLLO-B sugere um benefício potencial da patisirana na função cardíaca.

A cintilografia com marcador ósseo com 99m-Tecnécio é atualmente usada no diagnóstico não invasivo de ATTR-CM, devido à sua alta sensibilidade,^14^ mas o impacto da terapia na captação cardíaca desse biomarcador não é bem compreendido. Análises exploratórias em um subgrupo de pacientes brasileiros baseadas na captação cardíaca de tecnécio-99m mostraram que a escala de Perugini foi inalterada ou melhorou nos pacientes tratados com patisirana. Diversos pacientes mostraram uma melhora para o grau 1, que está abaixo do limiar para o diagnóstico de ATTR-CM por critérios não invasivos.^14^ Efeitos semelhantes de terapias de RNAi na captação cardíaca de tecnécio-99m foram relatados anteriormente em pacientes com hATTR com polineuropatia e hATTR com cardiomiopatia.^22,23^ Além disso, a redução na escala de Perugini foi descrita em raros pacientes com ATTR-CM com reversão espontânea associada a anticorpos e melhora clínica.^24^ Coletivamente, esses dados indicam um benefício de atuar na deposição amiloide cardíaca através da redução de TTR ou de mecanismos mediados pelo sistema imunológico. A relação entre a redução na captação miocárdica e os desfechos clínicos requer investigação adicional.

O perfil de segurança da patisirana em pacientes brasileiros no APOLLO-B foi semelhante ao da população geral do estudo. A incidência e os tipos de eventos adversos nos grupos patisirana e placebo também foram similares.

As opções de tratamento para pacientes com ATTR-CM no Brasil são limitadas. Tafamidis, que estabiliza os tetrâmeros de proteína TTR, era a única farmacoterapia aprovada para ATTR-CM até recentemente, quando a patisirana foi aprovada para esta condição. No estudo de Fase 3 ATTR-ACT, tafamidis reduziu a mortalidade por todas as causas e hospitalizações relacionadas ao sistema cardiovascular em comparação com o placebo^25^ e atenuou a taxa de declínio na função do ventrículo esquerdo (VE).^26^ Embora a taxa de declínio na capacidade funcional e qualidade de vida tenham sido reduzidas com tafamidis em comparação com o placebo, ambas continuaram a declinar durante o tratamento.^25^ Os achados da subpopulação brasileira do APOLLO-B sugerem que a rápida e sustentada redução da TTR com patisirana tem benefícios nos biomarcadores de estresse cardíaco e nos marcadores substitutos da carga de amiloide cardíaca, e que esses efeitos estão associados a benefícios potenciais na capacidade funcional, estado de saúde e qualidade de vida.

Os resultados apresentados devem ser interpretados no contexto das limitações relacionadas à análise post hoc e ao tamanho pequeno da amostra. O estudo não foi desenhado para detectar efeitos de tratamento específicos para a subpopulação brasileira. O APOLLO-B excluiu pacientes com a doença mais grave (ou seja, pacientes tanto na Classe III da NYHA quanto no estágio 3 da amiloidose ATTR ou na Classe IV da NYHA) e, portanto, os benefícios potenciais podem não se aplicar a todos os pacientes. Como nenhum paciente na subpopulação brasileira estava recebendo tafamidis no período basal, não foi possível avaliar a eficácia e segurança da patisirana com tafamidis concomitante. Finalmente, o período duplo-cego de 12 meses limita a interpretação do efeito da patisirana em desfechos como mortalidade e hospitalizações.

## Conclusões

Esta análise post hoc da subpopulação de pacientes brasileiros com ATTR-CM do estudo APOLLO-B mostrou uma rápida e sustentada redução da TTR. Demonstrou os efeitos da patisirana na capacidade funcional, estado de saúde e qualidade de vida, consistentes com as observações da população global do APOLLO-B, embora a significância não possa ser analisada devido ao tamanho pequeno da amostra. Além disso, foram observados benefícios potenciais na função cardíaca, incluindo uma mudança qualitativa na escala de Perugini de captação cardíaca durante a cintilografia com 99m-Tecnécio-pirofosfato, observada apenas em pacientes que foram submetidos ao tratamento com patisirana.
