# Supplementary material for: Tratamento com Patisirana na Subpopulação Brasileira do Estudo de Fase 3 APOLLO-B em Amiloidose por Transtirretina com Cardiomiopatia: Análise Post Hoc
Source: Arq Bras Cardiol. 2025 Apr 8;122(4):e20240568. [Article in Portuguese] doi: 10.36660/abc.2024568 (PMC12107767; doi:10.36660/abc.2024568)
Supplement: Translation

# Translation: Patisiran Treatment in the Brazilian Subpopulation of the Phase 3 APOLLO-B Study in Transthyretin Amyloidosis with Cardiomyopathy: Post Hoc Analysis

**DOI:** 10.36660/abc.20240568i

**Authors:** Claudio Tinoco Mesquita, Pedro Schwartzmann, Edileide Barros Correia, Marcus V. Simões, Andreia Biolo, Daniel Rodriguez Duque, Patrick Y. Jay, Fábio Fernandes

**Affiliations:** 1 Brazil Hospital Pró-Cardíaco, Rio de Janeiro, RJ – Brazil; 2 Brazil Universidade Federal Fluminense, Niterói, RJ – Brazil; 3 Brazil Unimed Ribeirão Preto, Ribeirão Preto, SP – Brazil; 4 Brazil Instituto Dante Pazzanese de Cardiologia, São Paulo, SP – Brazil; 5 Brazil Universidade de São Paulo Hospital das Clínicas da Faculdade de Medicina de Ribeirão Preto, Ribeirão Preto, SP – Brazil; 6 Brazil Universidade Federal do Rio Grande do Sul, Porto Alegre, RS – Brazil; 7 USA Alnylam Pharmaceuticals Inc, Cambridge, Massachusetts – USA; 8 Brazil Instituto do Coração do Hospital das Clínicas da Faculdade de Medicina da Universidade de São Paulo, São Paulo, SP – Brazil

**Keywords:** Amyloidosis, Transthyretin, Cardiomyopathies, Small Interfering RNA, Clinical Trial

## Abstract

**Background:**

Patisiran rapidly knocked down transthyretin and preserved functional capacity in patients with transthyretin amyloidosis with cardiomyopathy (ATTR-CM) in the global Phase 3 APOLLO-B study (NCT03997383).

**Objectives:**

To evaluate patisiran efficacy and safety in post hoc analysis of the Brazilian subpopulation of APOLLO-B.

**Methods:**

Patients were randomized 1:1 to patisiran 0.3 mg/kg or placebo every 3 weeks for 12 months. The primary endpoint was the change from baseline (CFB) in functional capacity (6-minute walk test [6MWT]) at Month 12. Secondary endpoints included CFB to Month 12 in the Kansas City Cardiomyopathy Questionnaire-Overall Summary (KCCQ-OS) score. Exploratory endpoints included CFB in cardiac biomarkers and Perugini grade of cardiac uptake during technetium-99m scintigraphy.

**Results:**

Forty-two patients enrolled in Brazil (patisiran, n=20; placebo, n=22). Patisiran showed benefit in 6MWT and KCCQ-OS scores vs. placebo; CFB (95% confidence interval [CI]) in 6MWT (median) and KCCQ-OS scores (least squares mean) was –2.0 m (–58.5, 42.9) and 9.37 (1.93, 16.81) points with patisiran vs. –30.1 m (–72.2, 3.5) and 2.62 (–4.68, 9.92) points for placebo. For cardiac biomarkers, the mean fold-change from baseline (95% CI) for N-terminal prohormone B-type natriuretic peptide and troponin I was 1.31 (1.06, 1.61) and 1.12 (0.94, 1.34) for patisiran, and 1.71 (1.39, 2.10) and 1.28 (1.08, 1.53) for placebo, respectively. Perugini grade improved in 11/18 (61.1%) and 0/10 evaluable patients with patisiran and placebo, respectively. There were no deaths in the patisiran group vs. 3 in the placebo group.

**Conclusion:**

The efficacy and safety of patisiran in Brazilian patients with ATTR-CM in APOLLO-B were consistent with those in the global study population. Findings are descriptive due to the small number of patients.

## Introduction

Transthyretin amyloidosis (ATTR) is a rare, rapidly progressive, debilitating, and potentially fatal disease.^1^ Misfolded transthyretin (TTR) forms toxic amyloid fibrils that deposit throughout the body, including the heart, nerves, gastrointestinal tract, and musculoskeletal system.^1-3^ ATTR can be hereditary (hATTR, also known as ATTRv), where patients inherit *TTR* variants that destabilize TTR protein, causing it to dissociate and misfold, or wild-type (wtATTR), where misfolded wild-type TTR accumulates as amyloid deposits in older adults.^1^ Clinically, hATTR presents as polyneuropathy, cardiomyopathy, or commonly both.^1,4,5^ Conversely, wtATTR manifests primarily as cardiomyopathy, but polyneuropathy may also be present.^2,5^

Data from the international Transthyretin Amyloidosis Outcomes Survey (THAOS) show that most symptomatic patients with ATTR in Brazil have neurologic and gastrointestinal system manifestations, and approximately 30% have cardiac involvement.^6^ Approximately 25% of Brazilian patients with symptomatic ATTR in the THAOS registry were misdiagnosed, and more than one-third experienced a delay in diagnosis of greater than 1 year, which may have resulted in a delay in starting treatment.^6^ Without intervention, the continuous accumulation of amyloid in the heart promotes the progression of cardiomyopathy and aggravates the associated cardiac manifestations.^7^ Consequently, ATTR with cardiomyopathy (ATTR-CM) has a severe and progressive course, placing affected individuals at high risk of cardiovascular-related hospitalization and death.^7^ Median survival after diagnosis in untreated patients is limited: 2.5 years for hATTR caused by the *TTR* Val122Ile (or pV142I) mutation and 3.6 years for wtATTR.^8^ The consensus guidelines of the *Sociedade Brasileira de Cardiologia* emphasize the importance of early diagnosis and treatment of ATTR-CM.^9^ However, the therapeutic options available for this condition are still limited.

Patisiran, an RNA interference (RNAi) therapeutic formulated in a lipid nanoparticle, targets hepatic *TTR* mRNA and rapidly knocks down circulating levels of both wild-type and variant TTR protein.^10^ Patisiran has been approved for the treatment of hATTR with polyneuropathy.^11,12^ Patisiran is not approved for the treatment of ATTR-CM in the USA, but it was recently approved in Brazil by the *Agência Nacional de Vigilância Sanitária* (ANVISA), and the *Agence nationale de sécurité du médicament et des produits de santé* has granted approval for the compassionate use of patisiran in France for patients with ATTR-CM failing tafamidis 61 mg. In the Phase 3 APOLLO-B study (NCT03997383), patisiran preserved functional capacity, health status, and quality of life vs. placebo over 12 months in patients with ATTR-CM (hATTR and wtATTR).^13^ Exploratory analyses also showed improvement in cardiac biomarkers and left ventricular (LV) structure and function compared with placebo.^13^ This post hoc analysis assessed the efficacy and safety of patisiran in the Brazilian subpopulation of APOLLO-B. We also assessed technetium-99m bone-tracer scintigraphy, a non-biopsy diagnostic technique for ATTR-CM, to better understand the impact of therapy on the cardiac uptake of this tracer.

## Methods

### Study design

APOLLO-B is an international, Phase 3, randomized, multicenter study of patisiran in patients with ATTR-CM (hATTR and wtATTR), comprising a 12-month, placebo-controlled, double-blind period and an ongoing open-label extension (OLE; NCT02510261). The study is being conducted in 21 countries across North America, Latin America, Europe, and Asia–Pacific. Full details of the study design have been published.^13^

In brief, patients were randomized 1:1 to receive patisiran (0.3 mg/kg up to a maximum of 30 mg) or placebo intravenously (IV) once every 3 weeks (Q3W) for 12 months. All patients in both treatment arms received standard premedication 60 minutes before infusion to reduce the potential for an infusion-related reaction with patisiran. Patients who completed the 12-month double-blind period were eligible to receive patisiran 0.3 mg/kg IV Q3W in the ongoing 36-month OLE period.

The study protocol and amendments were reviewed and approved by the Institutional Review Board or Independent Ethics Committee at each center. The study was conducted in accordance with all applicable regulatory requirements, the current guidelines of Good Clinical Practice, and principles originating from the Declaration of Helsinki. All patients provided written informed consent before participation.

### Patients

Full inclusion and exclusion criteria have been published.^13^ Patients were aged 18–85 years with a diagnosis of ATTR-CM (hATTR or wtATTR), defined as TTR amyloid deposition on tissue biopsy or fulfilling validated non-biopsy diagnostic criteria (i.e., Grade 2 or 3 cardiac uptake on technetium scintigraphy in patients who do not have monoclonal gammopathy);^14^ evidence of cardiac involvement by echocardiography, with an end-diastolic interventricular septal wall thickness >12 mm; and a medical history of heart failure. Patients were previously untreated with tafamidis or were permitted to have received tafamidis for ≥6 months at baseline as per the local label and approved dose, with disease progression as determined by the investigator.

Patients were excluded if classified as both New York Heart Association (NYHA) Class III and ATTR stage 3 (defined as *N*-terminal prohormone B-type natriuretic peptide [NT-proBNP] >3000 ng/L, concomitantly with an estimated glomerular filtration rate <45 mL/min/1.73 m^2^)^15^ NYHA Class IV; 6-minute walk test (6MWT) distance <150 m; polyneuropathy disability score >2; and other non-TTR cardiomyopathy.

### Endpoints

The primary endpoint was the change from baseline in functional capacity measured using the 6MWT at Month 12 for patisiran vs. placebo. Pre-specified secondary endpoints assessed in the Brazilian subpopulation included: change from baseline to Month 12 in the Kansas City Cardiomyopathy Questionnaire-Overall Summary (KCCQ-OS) and domain scores,^16^ composite endpoint of all-cause mortality, frequency of all-cause hospitalizations, and urgent heart failure visits over 12 months. Exploratory endpoints included change in cardiac biomarkers (NT-proBNP and troponin I) at Months 3, 6, 9, and 12, and Perugini grade assessed by cardiac uptake during technetium-99m scintigraphy with bone tracers. Technetium-99m scintigraphy was performed in a planned subset of patients. Pharmacodynamic effects were assessed by changes in serum TTR levels from baseline through Month 12. Safety outcomes, including adverse events (classified according to Medical Dictionary for Regulatory Affairs version 23.0 System Organ Class and Preferred Term), clinical laboratory parameters, and vital signs, were monitored throughout the study.

### Statistical analyses

For the primary endpoint, the median treatment effect with 95% confidence interval was estimated using the Hodges–Lehmann estimator; missing data were imputed as previously described.^13^ The median was used due to the non-normality of the 6MWT endpoint. Change from baseline at Month 12 in KCCQ-OS and component scores was assessed using a mixed-effects model for repeated measures (MMRM) adjusted for baseline KCCQ-OS as a continuous covariate and fixed-effect terms (treatment arm, visit, baseline tafamidis, subgroup [Brazil vs. ex-Brazil], treatment-by-visit interaction, treatment-by-subgroup interaction, visit-by-subgroup interaction, and treatment-by-visit-by-subgroup interaction). Change from baseline in cardiac biomarkers (NT-proBNP and troponin I) was assessed with an MMRM model, with log-transformed baseline values as a continuous covariate and the same fixed-effect terms as in the KCCQ-OS analysis. Sample size allowing, analyses followed the pre-specified models for subgroup analysis. MMRM models were fit using all trial participants, not just Brazilian participants, and it was the subgroup interactions within the models that permitted the estimation of specific effects for the Brazilian subpopulation.

A subset of patients in APOLLO-B participated in a planned technetium-99m single-photon emission computed tomography imaging study using bone-avid tracers. Perugini grade was evaluated at baseline and Month 12 (grade 0 = absent cardiac uptake; 1 = mild cardiac uptake less than bone; 2 = moderate cardiac uptake equal to bone or with mildly attenuated bone uptake; 3 = high cardiac uptake greater than bone or with a marked reduction in bone uptake).^14^ The change from baseline to Month 12 in Perugini grade was summarized descriptively.

Demographic and baseline characteristics, deaths, frequency of hospitalizations for any cause, urgent visits for heart failure in the overall population, and safety data were summarized descriptively. Events due to COVID-19 were excluded from the analysis. Efficacy and safety analyses were conducted in the Brazilian subpopulation of the full analysis set (i.e., all randomized patients who received any amount of the study drug) for the double-blind period of the study.

Pharmacodynamic analyses were conducted in all patients who received ≥1 complete dose of the study drug and had an evaluable baseline and ≥1 evaluable post-baseline serum TTR measurement. The percentage change from baseline in serum TTR levels was summarized descriptively.

All descriptive analyses sought to summarize continuous, normal variables through means and standard deviations, continuous, non-normal variables through medians and interquartile ranges (IQRs), and categorical variables through frequencies and percentages. Normality was assessed visually through histograms; data distributions that exhibited skewness or that contained outliers were deemed non-normal. SAS version 9.4 was used to perform the statistical analyses.

## Results

### Patients

A total of 360 patients were enrolled in APOLLO-B between October 2019 and May 2021. Of these, 42 patients were enrolled at 6 sites in Brazil, 39 of whom completed the Month 12 visit (Supplementary Materials Figure S1). The demographics and baseline clinical characteristics for the Brazilian subpopulation were generally comparable between treatment groups, except for numerically lower proportions of males and patients with wtATTR in the patisiran group compared with placebo (Table 1). Median (range) age was 73 (51–85) years in the Brazilian subpopulation. No patients were receiving tafamidis at baseline, but all patients were receiving concomitant cardiac medications (see Supplementary Materials Table S1 for details). Twenty-three (54.8%) patients had wtATTR, and 19 (45.2%) had hATTR. Among those with hATTR, there were 5 *TTR* variants, the most common being V122I (Table 1). A mixed phenotype with cardiomyopathy and polyneuropathy was reported in 17 (89.5%) patients with hATTR (see Supplementary Materials Table S1 for demographics and baseline clinical characteristics).

**Table 1:** – Baseline demographics and clinical characteristics

	Patisiran (n=20)	Placebo (n=22)
Age (years), median (IQR)*	73 (69–82)	73 (71–78)
**Male sex, n (%)**	14 (70.0)	20 (90.9)
**Race, n (%)^†^**		
White	12 (60.0)	16 (72.7)
Black/African American	6 (30.0)	5 (22.7)
Asian	0	1 (4.5)
Other	1 (5.0)	0
Not reported	1 (5.0)	0
**wtATTR, n (%)**	7 (35.0)	16 (72.7)
Diagnosed by biopsy^‡^	0	4 (25.0)
Diagnosed by technetium scintigraphy^‡^	7 (100.0)	15 (93.8)
**hATTR, n (%)**	13 (65.0)	6 (27.3)
Diagnosed by biopsy^‡^	2 (15.4)	2 (33.3)
Diagnosed by technetium scintigraphy^‡^	12 (92.3)	6 (100.0)
**hATTR with mixed phenotype of cardiomyopathy and polyneuropathy, n (%)^§^**	13 (100.0)	4 (66.7)
**TTR variants, n (%)^§^**		
I68L	0	1 (16.7)
T60A	3 (23.1)	1 (16.7)
V122I	9 (69.2)	3 (50.0)
V122L	0	1 (16.7)
V30M	1 (7.7)	0
**Time since diagnosis of ATTR (years), median (range)**	0.6 (0.1–1.8)	0.3 (0.1–1.6)
**Baseline tafamidis use, n (%)**	0	0
**ATTR stage, n (%)^¶^**		
1	13 (65.0)	12 (54.5)
2	7 (35.0)	8 (36.4)
3	0	2 (9.1)
**PND score, n (%)**		
0: no impairment	6 (30.0)	11 (50.0)
I: preserved walking, with sensory disturbances	13 (65.0)	11 (50.0)
II: impaired, walking without the need for a stick or crutches	1 (5.0)	0
**NYHA Class, n (%)**		
I	0	1 (4.5)
II	20 (100.0)	20 (90.9)
III	0	1 (4.5)
**6MWT distance (m), median (IQR)**	360.4 (323.8–379.1)	350.3 (300.0–434.7)
**KCCQ-OS (points), mean (SD)**	59.7 (22.4)	63.2 (23.2)
**NT-proBNP level (ng/L), median (IQR)**	1832.5 (904.5–3457.0)	2155.0 (1030.0–2911.0)
**Troponin I level (ng/L), median (IQR)**	64.2 (38.6–122.5)	78.0 (27.6–125.7)
**eGFR (mL/min/1.73m^2^), median (IQR)**	61.5 (54.5–72.5)	55.5 (39.0–72.0)
**Creatinine (μmol/L), median (IQR)**	101.5 (84.0–124.0)	115.0 (80.0–150.0)
**Medical history, n (%)**		
Diabetes mellitus	7 (35.0)	6 (27.3)
Hypertension	12 (60.0)	11 (50.0)
**Concomitant medications, n (%)**		
Diuretics	18 (90.0)	21 (95.5)
Mineralocorticoid receptor antagonists	13 (65.0)	11 (50.0)
Beta-blockers	5 (25.0)	11 (50.0)
ACEI, ARB, or ARNI	11 (55.0)	9 (40.9)
SGLT2 inhibitors	3 (15.0)	4 (18.2)

*Age at screening; ^†^Self-reported; ^‡^ATTR diagnosis could be confirmed by multiple methods; therefore, the sum of percentages may be greater than 100; ^§^Percentage based on number of patients with hATTR; ^¶^Stage 1 (lower risk) was defined by NT-proBNP level ≤3000 pg/mL and eGFR of ≥45 mL/min/1.73 m^2^. Stage 2 (intermediate risk) included all patients who did not meet the criteria for stages 1 or 3. Stage 3 (higher risk) was defined by NT-proBNP ≥3000 pg/mL and an eGFR of <45 mL/min/1.73 m^2^. 6MWT: 6-minute walk test; ACEI: angiotensin-converting enzyme inhibitor; ARB: angiotensin receptor blocker; ARNI: angiotensin receptor-neprilysin inhibitor; ATTR: transthyretin amyloidosis; eGFR: estimated glomerular filtration rate; hATTR: hereditary transthyretin amyloidosis; IQR: interquartile range; KCCQ-OS: Kansas City Cardiomyopathy Questionnaire-Overall Summary; NT-proBNP: N-terminal prohormone B-type natriuretic peptide; NYHA: New York Heart Association; PND: polyneuropathy disability; SD: standard deviation; SGLT2: sodium-glucose co-transporter 2; TTR: transthyretin; wtATTR: wild-type transthyretin amyloidosis.

### Efficacy

#### Primary endpoint

Patisiran-treated patients in the Brazilian subpopulation showed a smaller magnitude of decline from baseline in the 6MWT distance at Month 12 vs. placebo (Figure 1A).

**Figure 1:** – Change from baseline over time for (A) 6MWT and (B) KCCQ-OS. 6MWT: 6-minute walk test; CI: confidence interval; H–L: Hodges–Lehmann; KCCQ-OS: Kansas City Cardiomyopathy Questionnaire-Overall Summary; LS: least squares; SEM: standard error of the mean.

#### Secondary endpoints

KCCQ-OS scores increased from baseline to Month 12 with patisiran vs. placebo (Figure 1B). Component analyses favored patisiran vs. placebo for all domains (physical limitation, total symptom, quality of life, and social limitation) (Supplementary Materials Figure S2). A similar proportion of patients in the patisiran and placebo groups had ≥1 composite event (all-cause mortality or hospitalization or urgent heart failure visits; Table 2). There were no deaths in the patisiran group (Table 2).

**Table 2:** – All-cause deaths, all-cause hospitalizations, or urgent heart failure visits*

	Patisiran (n=20) PY=20.9	Placebo (n=22) PY=21.6
**Patients with ≥1 event^†‡^, n (%) [n events]**	6 (30.0) [10]	6 (27.3) [11]
**All-cause deaths, n (%) [n events]**	0	3 (13.6) [3]
Cardiovascular-related deaths	0	2 (9.1) [2]
Non-cardiovascular-related deaths	0	1 (4.5) [1]
**All-cause hospitalizations, n (%) [n events]**	5 (25.0) [9]	6 (27.3) [8]
Cardiovascular hospitalizations	3 (15.0) [4]	4 (18.2) [5]
Non-cardiovascular hospitalizations	2 (10.0) [5]	3 (13.6) [3]
**Urgent heart failure visits, n (%) [n events]**	1 (5.0) [1]	0

*Follow-up time computed using the last day in a 12-month double-blind period for efficacy analysis; ^†^Composite endpoint of all-cause death, all-cause hospitalizations, or urgent heart failure visits; ^‡^Events due to COVID-19 were excluded. PY: patient-years.

#### Exploratory endpoints

Median (IQR) change from baseline in NT-proBNP at Month 12 was 320.0 (3.5–1756.0) ng/L with patisiran and 2024.0 (–211.0 to 3231.7) ng/L with placebo. The ratio (patisiran:placebo) of adjusted geometric mean fold-change from baseline was larger in the placebo group than in the patisiran group (Figure 2A). For troponin I, a median (IQR) change from baseline of 8.5 (–9.10 to 23.4) ng/L was reported at Month 12 for the patisiran group compared with 16.5 (0–61.5) ng/L for placebo. The ratio (patisiran:placebo) of adjusted geometric mean fold-change from baseline was larger in the placebo group than in the patisiran group (Figure 2B).

**Figure 2:** – Adjusted geometric mean fold-change from baseline in (A) NT-proBNP and (B) troponin I. CI: confidence interval; NT-pro-BNP: N-terminal prohormone B-type natriuretic peptide

Thirty-five patients (patisiran, n=18; placebo, n=17) in the Brazilian subpopulation were included in the imaging subset. Twenty-eight patients (patisiran, n=18; placebo, n=10) were evaluable, with Perugini grading at both baseline and Month 12; 7 patients in the placebo group were not evaluable at Month 12. An improvement in Perugini grade at Month 12 was observed in 11 (61.1%) patients in the patisiran group. Of these 11 patients, 3 improved to Perugini grade 1. None of the placebo-treated patients showed an improvement in Perugini grade. No patients in the patisiran group showed an increase in Perugini grade, whereas 1 patient (10.0%) in the placebo group showed an increase in grade at Month 12. Patient-level data for change from baseline in Perugini grade for patients in the Brazilian subpopulation and those outside Brazil are shown in Figure 3A, and representative images are shown in Figure 3B.

**Figure 3:** Individual patient-level data for patients from Brazil and ex-Brazil for (A) Perugini grade at baseline and Month 12, and (B) representative technetium-99m cardiac scintigraphy images for an individual patient showing a reduction in myocardial uptake of tracer at 5 and 24 months after patisiran initiation (technetium-99m analysis set).

#### Pharmacodynamics

A rapid and sustained knockdown of serum TTR was observed in patients treated with patisiran (Figure 4). At Month 12, patisiran treatment led to a mean (standard error of the mean) percent reduction in serum TTR of 85.3% (4.5).

**Figure 4:** – Percentage change from baseline in serum TTR. Serum TTR levels were analyzed as previously described.13 SEM: standard error of the mean; TTR: transthyretin.

## Safety

A similar proportion of patients from Brazil in the patisiran and placebo groups had adverse events during the 12-month double-blind period (Table 3), with the majority being mild or moderate in severity and deemed unrelated to study treatment by investigators. The most frequent adverse event was cardiac failure (Table 3). No patients from Brazil had infusion-related reactions. Serious adverse events are shown in Supplementary Materials Table S3. No adverse events resulted in discontinuations, and 3 deaths occurred, all in the placebo group.

**Table 3:** – Overview of adverse events over the 12-month double-blind treatment period

Event, n patients (%)	Patisiran (n=20) PY=20.9^*^	Placebo (n=22) PY=21.5^*^
**≥1 AE (all-cause)**	19 (95.0)	21 (95.5)
**Treatment-related AEs**	2 (10.0)	2 (9.1)
**Most common AEs (all-cause)^†^**		
Heart failure	5 (25.0)	7 (31.8)
Renal impairment	3 (15.0)	2 (9.1)
Muscle spasms	3 (15.0)	1 (4.5)
COVID-19	3 (15.0)	2 (9.1)
Constipation	2 (10.0)	4 (18.2)
Nausea	2 (10.0)	0
Urosepsis	2 (10.0)	0
Autonomic nervous system imbalance	2 (10.0)	0
Insomnia	2 (10.0)	3 (13.6)
Hematuria	2 (10.0)	1 (4.5)
Atrial fibrillation	1 (5.0)	3 (13.6)
**SAEs (all-cause)**	8 (40.0)	7 (31.8)
**Treatment-related SAEs**	0	0
**Cardiac AEs (SOC)**	7 (35.0)	10 (45.5)
**Cardiac SAEs**	3 (15.0)	4 (18.2)
Atrial fibrillation	0	1 (4.5)
Cardiac failure	2 (10.0)	4 (18.2)
Tachyarrhythmia	1 (5.0)	0
**Severe AEs**	4 (20.0)	5 (22.7)
**AEs leading to treatment discontinuation**	0	0
**Treatment-related AEs leading to treatment discontinuation**	0	0
**Deaths**	0	3 (13.6)

AEs coded using MedDRA version 23.0. *Determined using last dosing day in the 12-month period; ^†^≥10% for Preferred Terms in either treatment arm. AE: adverse event; MedDRA: Medical Dictionary for Regulatory Affairs; PY: patient-years; SAE: serious adverse event; SOC: system organ class.

## Discussion

This post hoc analysis assessed the efficacy and safety of patisiran in the Brazilian subpopulation of the APOLLO-B study in patients with ATTR-CM (hATTR and wtATTR). Although the results are descriptive because the study was not powered to detect treatment effects specific to this subgroup, they are consistent with the overall findings of APOLLO-B. Rapid and sustained knockdown of circulating TTR by patisiran resulted in potential improvement in functional capacity, health status, and quality of life during the 12-month double-blind period compared with placebo and was well tolerated in Brazilian patients with ATTR-CM. A potential benefit on cardiac biomarkers and myocardial uptake in bone-avid tracer scintigraphy (Perugini grade) was also observed.

A clinically meaningful difference was evident with patisiran in terms of preserving functional capacity, with the observed magnitude of decline in 6MWT distance in the patisiran group being similar to that expected with healthy aging.^17,18^ A trend toward improvement in health status and quality of life, based on a moderate-to-large difference between the patisiran and placebo groups in the change from baseline in KCCQ-OS scores at Month 12, was also evident. Patisiran had a notable effect on symptoms, with a difference from placebo of approximately 12 points on the KCCQ symptom domain score and a corresponding impact on quality-of-life domains. These effects are of relevance to patients with ATTR in clinical practice who frequently report debilitating symptoms that negatively affect their daily lives and emotional well-being.^19^

Some differences were evident in the outcomes in the Brazilian subpopulation compared with the overall study population. Among patisiran-treated patients, KCCQ-OS scores did not increase in the overall APOLLO-B population to the same extent as seen in the Brazilian subpopulation. Placebo-treated patients in Brazil appear to show a less rapid decline in functional capacity than patients in the overall study population. In addition, health status and quality of life were maintained in the placebo group of the Brazilian subpopulation, while there was a decrease (worsening) in scores among the placebo-treated patients in the overall study. The reason for these differences is unknown but may relate to general heart failure management or a placebo effect.

In addition to its effects on functional capacity, health status, and quality of life, patisiran treatment resulted in a lower increase in the cardiac biomarkers NT-proBNP and troponin I compared with placebo. NT-proBNP and troponin I are used to assess cardiac manifestations in patients with ATTR-CM, and elevated levels are associated with more severe cardiac involvement and poorer cardiac and mortality outcomes.^20,21^ The improvement in levels of these cardiac biomarkers observed in Brazilian patients with ATTR-CM in APOLLO-B suggests a potential benefit of patisiran on cardiac function.

Technetium-99m bone-avid tracer scintigraphy is currently used in the non-biopsy diagnosis of ATTR-CM, owing to its high sensitivity,^14^ but the impact of therapy on the cardiac uptake of this tracer is not well understood. Exploratory analyses in a subset of Brazilian patients based on technetium-99m cardiac uptake showed that Perugini grade was either unchanged or improved in patients treated with patisiran. Several patients showed an improvement to grade 1, which is below the threshold for diagnosis of ATTR-CM by non-biopsy criteria.^14^ Similar effects of RNAi therapeutics on technetium-99m cardiac uptake have been reported previously in patients with hATTR with polyneuropathy and hATTR with cardiomyopathy.^22,23^ In addition, reduction in Perugini grade has been described in rare patients with spontaneous, antibody-associated reversal of ATTR-CM and clinical improvement.^24^ Collectively, these data indicate a benefit of targeting cardiac amyloid deposition by TTR knockdown or immune-mediated mechanisms. The relationship between reduction in myocardial uptake and clinical outcomes requires further investigation.

The safety profile of patisiran in Brazilian patients in APOLLO-B was similar to that in the overall study population. The incidence and types of adverse events in the patisiran and placebo groups were similar as well.

Treatment options for patients with ATTR-CM in Brazil are limited. Tafamidis, which stabilizes TTR protein tetramers, was the only pharmacotherapy approved for ATTR-CM until recently when patisiran was approved by ANVISA for this condition. In the Phase 3 ATTR-ACT study, tafamidis reduced all-cause mortality and cardiovascular-related hospitalization vs. placebo^25^ and attenuated the rate of decline in LV function.^26^ While the rate of decline in functional capacity and quality of life was reduced with tafamidis vs. placebo, both continued to decline during treatment.^25^ The findings from the APOLLO-B Brazilian subpopulation suggest that rapid and sustained knockdown of TTR with patisiran has benefits on biomarkers of cardiac stress and surrogate markers of cardiac amyloid burden, and that these effects are associated with potential benefits on functional capacity, health status, and quality of life.

The presented results should be interpreted in the context of limitations related to post hoc analysis and small sample size. The study was not designed to detect treatment effects specific to the Brazilian subpopulation. APOLLO-B excluded patients with the most severe disease (i.e., patients in both NYHA Class III and ATTR amyloidosis stage 3 or in NYHA Class IV), so potential benefits may not apply to all patients. Because no patients in the Brazilian subpopulation were receiving tafamidis at baseline, it was not possible to assess the efficacy and safety of patisiran with concomitant tafamidis. Finally, the 12-month double-blind period limits the interpretation of the effect of patisiran on outcomes such as mortality and hospitalizations.

## Conclusions

This post hoc analysis of the subgroup of Brazilian patients with ATTR-CM from APOLLO-B showed rapid and sustained knockdown of TTR. It demonstrated the effects of patisiran on functional capacity, health status, and quality of life, consistent with observations from the global APOLLO-B population, although significance could not be analyzed due to the small sample size. Additionally, potential benefits on cardiac function were observed, including a qualitative change in Perugini grade of cardiac uptake during technetium-99m scintigraphy that was noted only in patients who underwent patisiran therapy.
